# Alterations in the brain lipidome of Alzheimer's disease donors with rare *TREM2* risk variants

**DOI:** 10.1093/braincomms/fcaf452

**Published:** 2026-01-21

**Authors:** Petroula Proitsi, Amera Ebshiana, Asger Wretlind, Jin Xu, Angela K Hodges, Cristina Legido-Quigley

**Affiliations:** Centre for Preventive Neurology, Wolfson Institute of Population Health, Queen Mary University of London, London EC1M 6BQ, UK; Maurice Wohl Clinical Neuroscience Institute, Institute of Psychiatry, Psychology and Neuroscience (IoPPN), King’s College London, London SE5 9RT, UK; Institute of Pharmaceutical Science, King’s College London, London SE1 9NH, UK; Institute of Pharmaceutical Science, King’s College London, London SE1 9NH, UK; Institute of Pharmaceutical Science, King’s College London, London SE1 9NH, UK; Maurice Wohl Clinical Neuroscience Institute, Institute of Psychiatry, Psychology and Neuroscience (IoPPN), King’s College London, London SE5 9RT, UK; Institute of Pharmaceutical Science, King’s College London, London SE1 9NH, UK; Clinical Research, Steno Diabetes Center Copenhagen, Herlev 2730, Denmark

**Keywords:** *TREM2*, Alzheimer's disease, brain, lipids, lipidomics

## Abstract

Triggering Receptor Expressed on Myeloid Cells 2 (TREM2) is a microglial receptor, sensitive to Phospholipids and Sphingomyelins, associated with neurodegeneration. Hypomorphic variants in the *TREM2* gene significantly increase the risk of developing Alzheimer’s disease (AD). The aim of this study was to characterize networks of lipids in post-mortem brain tissue from AD and Control donors, and to identify lipids associated with AD and impacted by dysfunctional TREM2. We studied human post-mortem brain tissue from the hippocampus and Brodmann area 9 (BA9) from 102 brains. Brain tissue from BA9 was available from *n* = 55 donors (14 Ad donors with a non-synonymous *TREM2* risk variant [AD(*TREM2*+)], 20 Ad donors with no *TREM2* risk variants [Ad(*TREM2*−)] and 21 Control donors), and brain tissue from the Hippocampus was available for *n* = 47 brain donors (7 Ad[*TREM2*+], 20 Ad[*TREM2*−] and 20 Control donors). Mass Spectrometry was performed to obtain lipidomic signatures spanning 99 lipid species that included the following lipid classes: Ceramides, Sphingomyelins, Phosphatidic acids, Phosphatidyl-cholines, Phosphatidyl-ethanolamines, Phosphatidyl-glycerols, Phosphatidyl-inositols, Phosphatidyl-serines and Triglycerides. Weighted gene co-expression network analysis (WGCNA) was used to identify highly correlated lipid modules and hubs in each brain region. Generalized least squares and linear regression analyses, adjusted for age at death, biological sex, number of Apolipoprotein E (*APOE*) ε4 alleles, and post-mortem delay, were used to assess the associations of lipid modules and hubs with AD and *TREM2*, in combined analyses across regions and in each region separately. Four lipid modules were relatively well-preserved between the two brain regions, and three of these modules were altered in AD donors and/or in AD *TREM2* carriers. Levels of the BA9 ‘turquoise’ module (‘blue’ hippocampus module), enriched in Sphingolipids and Phospholipids, were elevated in AD donors and particularly in AD *TREM2* carriers [AD(*TREM2*+)]. The hub lipid of the BA9 ‘turquoise’/hippocampus ‘blue’ module, Phosphatidyl-serine [PS(32:1)], was increased in AD versus Control donors (beta = 0.677, 95% CI 0.28–1.08, *P* = 1.14E−03), and in AD(*TREM2*+) versus Control donors (beta = 1.00, 95% CI 0.53–1.48, *P* = 5.57E−03), whereas the strongest association was observed with Ceramide [Cer(d38:1)] increased in AD versus Control donors (beta = 0.929, 95% CI 0.46–1.40, *P* = 1.67E−04) and in AD(*TREM2*+) versus Controls donors (beta = 1.31, 95% CI 0.78–1.84, *P* = 4.35E−06). The consistent increase in TREM2 ligands such as Ceramides and Phosphatidyl-serines in the brains of AD donors, particularly *TREM2* risk variants carriers, could reflect the presence of AD-associated damage signals in the form of stressed or apoptotic cells and damaged myelin.

## Teaser

Proitsi *et al*. report that lipidomic network analysis of post-mortem brain tissue reveals increased levels of phospholipids and sphingomyelins in Alzheimer’s disease brain donors, especially in carriers of rare *TREM2* risk variants. These findings further implicate *TREM2* in lipid regulation providing insights into lipid-mediated mechanisms in Alzheimer’s disease.

## Graphical Abstract

**Graphical Abstract:**
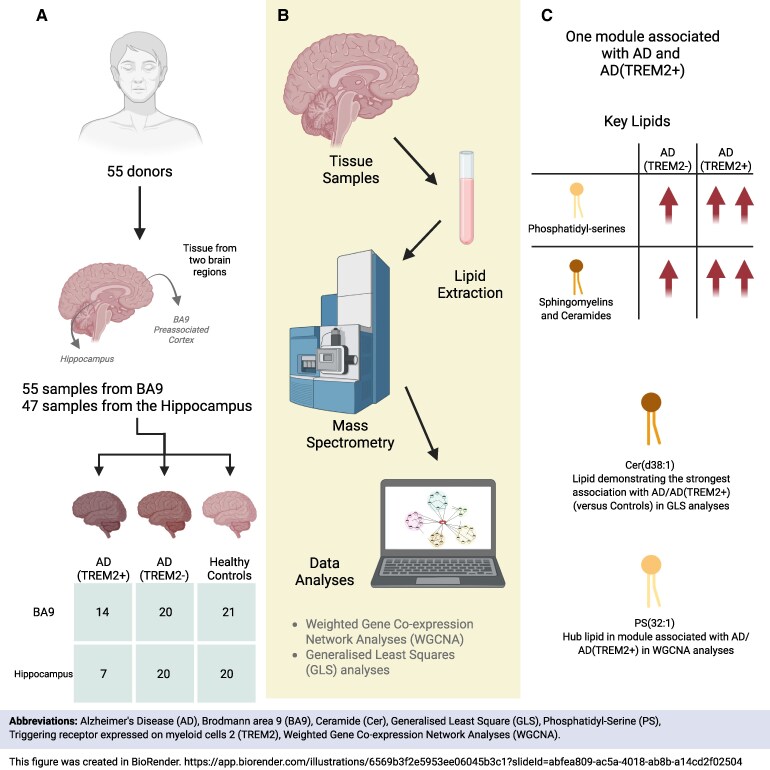

## Introduction

Life expectancy has steadily increased in recent years, but with it brings greater prevalence of age-related conditions such as Alzheimer’s disease (AD), the most common cause of dementia. Dementia numbers are predicted to grow from 46.8 M to 131.5 M by 2050^1^ highlighting the urgent need for effective treatments. DNA variants linked to ∼84 genes are consistently associated with AD risk, of which ∼25% have highly enriched or specific expression in brain microglia (reviewed in^2^). Genes with immune or lipid function have been recognized for some time to be enriched in AD GWAS results.^3^ One of these genes *TREM2*, codes for the Triggering receptor expressed on Myeloid Cells 2. Rare (MAF <1%), but large-impact hypomorphic variants in *TREM2* significantly increase AD risk^4-7^ and related conditions.^8-12^ Rare recessive *TREM2* mutations can also cause Nasu-Hakola disease characterized by fragility fractures, brain white matter changes and dementia in early adulthood.^13^

Most *TREM2* risk variants cluster in the extracellular Ig-like V-type domain impacting production (rs104894002, Q33X) expression or turnover of *TREM2* at the cell surface (rs75932628, R47H, MAF = 0.02),^14,15^ α-secretase cleavage of the extracellular ectodomain (s*TREM2* production) (rs2234255, H157Y),^15–17^ shedding of s*TREM2* (rs75932628, R47H)^18^ and/or ligand binding (rs75932628, R47H; rs143332484, R62H; rs2234253/rs2234258/rs2234256, T96 K/W191X/L211P; rs2234255, H157Y).^19–21^

TREM2 is a damage-response receptor expressed exclusively by myeloid cells including brain microglia.^22^ It has a preference for binding anionic lipids, notably Phosphatidyl-serine (PS)^21,23–25^ a membrane signal on apoptotic cells, notably neuronal synapses in AD.^26^ Other lipids reported to activate TREM2 include Phosphatidyl-ethanol (PE), Phosphatidyl-choline (PC), Phospholipids and Sphingolipids such as Ceramides (Cer) and Sphingomyelins (SM). Damaged oligodendrocyte myelin and stressed or apoptotic cells expose sphingolipids in AD.^19-21,23,24,27-31^ APOE and other apolipoproteins and Aβ oligomers also appear to be ligands for TREM2.^20,32-36^

TREM2-related pathologies include regional brain atrophy, myelin loss, swollen axons in white matter and changes the density and shape of microglia subsets.^37,38^ TREM2 dysfunction prevents microglia from switching to a glycolytic state^39^ resulting in deficits in Aβ, myelin debris, apoptotic neuron and *E.coli* phagocytosis.^15,40-43^ Results from analysis of very rare *TREM2* risk cases are limited. Nevertheless, non-hydroxy fatty acids of sulfatide (C16–C18) were found to be higher in Nasu-Hakola cortex compared to matched controls, while longer-chain (C24) fatty acids were lower.^44^ Additionally, free fatty acids were increased^45^ while cholesterol and Cer in white matter were reduced.^46^ Microglia from a cuprizone demyelinating TREM2-deficient mouse model were found to still sense and take up myelin debris but failed to efflux myelin cholesterol, resulting in cellular cholesteryl ester (CE) accumulation.^25^ In that same study, shorter fatty acid Cer were elevated indicative of inflammation. Together, TREM2 deficiency appears to lead to broad lipid dysregulation.

In AD cases with a *TREM2* risk variant, plaques are more diffuse and nearby microglia have reduced membrane ruffling, and shorter but more numerous filopodia when stimulated with ATP or M-CSF.^47^ Loss of TREM2 impairs actin ring formation and podosomes in osteoclasts, essential for phagocytosis linked to bone resorption.^48^ PIP2 to PIP3 conversion at the cell membrane is essential for ‘sealing off’ the membrane edges during motility, endocytosis, exocytosis and phagocytosis through the cytoskeletal system. TREM2 signaling links to this pathway through Syk, MAPK, PIP2-PI3K-PIP3 Rac1 and Cdc42 involving lipid species.^31,49^

Over-expression, activation or restoration of normal TREM2 function has largely beneficial outcomes in amyloidogenic mouse models^50-53^ while knock-out or haploinsufficiency exacerbates amyloid-associated pathologies.^38,54-57^ These benefits are likely linked to improved phagocytosis of amyloid and clearance of damaged neurons by phagocytosis. It is noteworthy that amyloid plaques contain various lipids [Sphingolipids such as Cer, Phospholipids, Lysophospholipids as well as Cholesterol and Triglycerides (TGs)] which may themselves influence pathogenesis.^58-60^ Cer are potent inflammatory signaling molecules associated with AD in circulation.^61^

We hypothesize brain lipid dysregulation in AD reflects unresolved pathologies (damaged myelin, stressed/dying neurons and their knock-on impacts on lipid metabolism and membrane composition) exacerbated in people with *TREM2* risk variants where microglia fail to recognize and clear these pathologies effectively. Here, we sought to characterize networks or modules of highly connected lipids in post-mortem brain tissue from AD and control donors, and to identify key lipid ‘hubs,’ i.e. highly connected lipids likely to play central roles in the functioning and regulation of these networks, associated with AD and impacted by *TREM2*. Specifically, we used a network approach used traditionally for genomic data, WGCNA, in post-mortem brain from two areas, the Hippocampus (HC) and the BA9 pre-association cortex. We investigated whether these networks and their ‘hubs’ were altered in brain tissue from AD donors compared to control donors, and in brain tissue from AD carriers of rare *TREM2* risk variants [AD(*TREM2*+)] compared to controls and AD donors with no *TREM2* risk variants [AD(*TREM2*−)]. Results from this study highlight lipid networks of highly correlated lipids and key ‘hubs’ connected to AD pathology. It also provides insights into the role of lipids in *TREM2*-mediated activation of microglia in AD, which together highlight processes to target in future therapeutic strategies.

## Materials and methods

### Study participants and samples

Informed consent for all brain donors was obtained according to the Declaration of Helsinki (1991) and protocols and procedures were approved by the relevant ethical committee and by each brain bank. Brain tissue was provided following project approval by the London Neurodegenerative Diseases Brain Bank (LNDBB). LNDBB subjects were approached in-life for written consent for brain banking, and all tissue donations were collected, stored and distributed following legal and ethical approval (Wales REC 3 favorable opinion 17 Sep 2013, REC number 08/MRE09/38+5; LBBND HTA license number 12293).

Pathological diagnosis was made according to established methods at the time of donation.^62-66^ Where necessary for historical cases, retrospective diagnoses were made using current criteria. Post-mortem human brain tissue, from the HC and BA9 pre-association cortex was initially obtained from a total of *n* = 60 brain donors: 34 Ad donors and 26 control donors. Tissue from the BA9 pre-association cortex was available for all 60 donors, tissue from the HC was available for 49 donors.

### *TREM2* genetic variants

*TREM2*+ cases were identified in a number of ways. (i) sequencing *TREM2* Exon 2 in 631 Ad and normal elderly control brain donors from the MRC LNDBB (King’s College London, UK) (9 *TREM2*+ donors identified); (ii) sequencing *TREM2* Exon 2 in 198 Ad cases from The Netherlands Brain Bank (Royal Netherlands Academy of Arts and Sciences, Netherlands) (4 *TREM2*+ donors identified); (iii) through a collaboration with Queen Square Brain Bank for Neurological Disorders (University College London, UK) using previously published cases^4^ (6 *TREM2*+ donors identified). From these 19 *TREM2*+ donors, 14 had a pathologically confirmed diagnosis of AD and were included in further analyses. The remaining five donors were pathologically normal at death, although one of them displayed symptoms of mild cognitive impairment just prior to death and was subsequently assessed as Braak stage III at post-mortem, and were excluded from further analyses. This left 55 donors from three groups: 14 Ad(*TREM2*+) donors (AD donors with a non-synonymous DNA variant in *TREM2* expected to be at higher risk of AD), 20 Ad(*TREM2*−) donors (AD donors with no-disease associated variant) and 21 Control donors (tissue from age-matched donors with no disease-associated *TREM2* variant or AD pathology). Half of the 14 donors in the AD(*TREM2*+) group (*n* = 7) had the established *TREM2*  Ad risk variant c.140G>A; p.R47H (rs75932628).^5,67-72^ The remaining *n* = 7 Ad(*TREM2*+) donors had one of five additional very rare non-synonymous *TREM2* variants either with functional evidence and/or predictive to be pathogenic in simulation programs: p.Q33X (rs104894002),^20,73^ p.D39E (rs200392967),^67,74^ p.G58A (rs886042808), p.D87N (rs142232675)^68^ or haplotype p.T96K/p.W191X/p.L211P (rs2234253/rs2234258/rs2234256)^19,20,75^ (Table 1).

**Table 1:** Donor and sample characteristics

	BA9 (*n* = 55)				HC (*n* = 47)			
Diagnosis, (*n*)	AD (TREM2+), (*n* = 14)	AD (TREM2−), (*n* = 20)	Controls, (*n* = 21)	Χ^2^ or ANOVA	AD (TREM2+), (*n* = 7)	AD (TREM2−), (*n* = 20)	Controls, (*n* = 20)	Χ^2^ or ANOVA
Females/Males [% Female]	8/6 (57%)	12/8 (60%)	8/13 (38.1%)	Χ^2^ = 2.26, df = 2, *P* = 0.323	4/3 (57%)	12/8 (60%)	8/12 (40%)	Χ^2^ = 1.72, df = 2, *P* = 0.423
Age (years), mean (sd)	69.64 (14.6)	77.75 (10.4)	72.4 (12.6)	F(2,52) = 1.933, *P* = 0.155	73 (15.9)	77.8 (10.4)	72.7 (12.9)	F(2,44) = 0.946, *P* = 0.396
APOE ε4 alleles, *n* (0/1/2)	3/8/3	3/13/4	17/3/1	Χ^2^ = 21.59, df = 4, *P* = 2.42e−04	1/5/1	3/13/4	16/3/1	Χ^2^ = 20.163, df = 4, *P* = 4.64e−04
PMD (hours), mean (sd)	26.43 (21.9)	36.85 (22.4)	32.81 (18.9)	F(2,52) = 1.01, *P* = 0.370	19.7 (13.6)	36.85 (22.4)	32.4 (19.3)	F(2,44) = 1.887, *P* = 0.164
TREM2 variants breakdown (MAF):				NA				NA
p.R47H (c.140G > A, rs75932628) (MAF = 0.003)	7	0	0		4	0	0	
p.T96 K/p.W191X/p.L211P (rs2234253/rs2234258/rs2234256) (MAF = 1.2 × 10-4/0.002/0.01)	1	0	0		1	0	0	
p.D87N (rs142232675) (MAF = 0.001)	3	0	0		1	0	0	
p.D39E/(rs200392967) (MAF = 5.3 × 10^−5^)	1	0	0		0	0	0	
p.Q33X (rs104894002) (MAF = 3.6 × 10^−5^)	1	0	0		1	0	0	
p.G58A (rs886042808) (MAF = 1.6 × 10^−6^)	1	0	0		0	0	0	

Chi-squared (χ²) tests or one-way ANOVA were used to compare frequencies or means, respectively, between AD (TREM2+), AD (TREM2−), and control donors. Minor allele frequencies (MAFs) were obtained using the gnomAD browser (https://gnomad.broadinstitute.org/).

Abbreviations: ANOVA = One-way Analysis of Variance; BA9 = Brodmann area 9 pre-association cortex; HC = Hippocampus; MAF = Minor Allele Frequency; PMD = Post-mortem delay.

A total of 55 donors had tissue available from the BA9 pre-association cortex and 47 had tissue available from the HC (summarized in the Graphical Abstract and in Table 1).

### Sample preparation

Samples were randomized and lipids extracted from 30 mg of frozen tissue based on an in-vial dual extraction protocol.^76^ Briefly, 10 μl of water was added to 50 μl of the homogenate. Vials were then vortexed for 5 min, after which 250 μl of methyl-tertiary butyl ether (MTBE) containing Tripentadecanoin (10 μg/ml) and Heptadecanoic acid (10 μg/ml) was added, and samples were again vortexed at room temperature for 60 min. Following the addition of a further 40 μl of water containing 0.15 mM ammonium, samples were centrifuged at 2500×g for 30 min at 4°C. This resulted in clear separation of an upper MTBE and lower aqueous phase.

### Data acquisition

LC-MS Lipidomics analysis of the upper MTBE layer in positive mode was performed on a Waters Acquity ultra performance liquid chromatogram (UPLC) system coupled to a Waters Premier quadrupole time-of-flight (Q-Tof) mass spectrometer (Waters, Milford, MA, USA). Briefly, 5 μl of sample extract was injected onto an Agilent Poroshell 120 EC-C8 column (150 mm × 2.1 mm, 2.7 μm). The gradient started at 80% mobile phase B increasing linearly to 96% B in 23 min and was held until 45 min then the gradient was increased to 100% by 46 min until 49 min. Initial conditions were restored in 2 min ahead of 7 min of column re-equilibration. Data were collected in the centroid mode over the mass range m/z 50–1000 with an acquisition time of 0.1 secondsper scan. Samples were analysed in a randomized order along with pooled brain samples (Quality control samples) after every eight injections.

### Data pre-processing and lipid identification

All data was collected by Waters Xevo QTOF which used a MSe technique. Two collision energies were applied enabling data collection at two levels. The first level obtained data using 5 V of collision energy, the second level obtained data at a higher collision energy of 50 V. This can assist in structural elucidation and to simultaneously collect accurate mass of parent ion and fragmentation data. Following LC-MS analysis of samples, the MS raw data were transformed into mzXML format using msConvert (ProteoWizard). XCMS software package in R was then used to analyse the converted mzXML data files, which underwent preprocessing steps of peak picking and alignment processed, using a ‘centwave’ method which enables the deconvolution of closely eluting or slightly overlapping peaks. Data was normalized to total peak area, imputed and inverse variance transformed.

A total of 99 lipids were identified in the brain samples following our previous established protocol.^77^ Identification was based on fragmentation patterns and comparison with lipid features from our in-house lipid library containing pure standards. Lipid species were examined in positive ion mode and the product ions of the [M+H]^+^ precursor was used to determine their acyl composition. As an example, the fragment ion 184.03 Da corresponds to phosphocholine head group. Twenty-six Sphingolipids (16 SM and 10 Cer) were identified with a parent molecular ion in the form [M−H2O+H]^+^ and the fragment ion 264.26 Da which corresponds to a Sphingosine base chain. We named the fatty acid chains as very long (VLCH), longer (LCH) or shorter (SCH), but these names are only for reference in this manuscript, as there isn't a single universal standard specifically for lipid naming based on chain length.

### Statistical analysis

#### Data quality control

Lipid features missing in ≥20% of donors, and donors with ≥20% missing features were excluded from further analyses. For the remaining data, missing data points (*n* = 4) were imputed using k-nearest neighbors (knn, k = 10) (‘impute’) and the dataset was subsequently inverse-normal-transformed.

#### Preliminary associations between lipids, brain regions and covariables

In preliminary analyses, we investigated the association of each lipid with sex, age (at death), number of APOEε4 alleles and post-mortem delay, combining the two brain regions (Supplementary Table 1). Linear model analysis was performed with lipids as outcomes using generalized least squares (GLS) (‘gls’ function in nlme R package^78^), which allows for a fully unstructured residual variance-covariance matrix. Analyses were initially performed separately for each covariable, adjusting for post-mortem delay with sex, age (at death), number of APOEε4 alleles as predictors. An interaction term between brain region and each predictor, was included in each model to investigate possible brain region-specific associations. In the presence of an interaction, linear regression analyses were repeated, stratifying for each brain region. To correct for multiple testing and the high correlation between some lipids (Supplementary Fig. 1), we set a Bonferroni metabolome-wide statistical significance threshold of *P* < 0.0011; the *P* < 0.05 significance level was divided by the number of principal components (*n* = 45) that explained over 95% of variation in the lipidomic data.

#### Preliminary associations between lipids, AD and *TREM2*

We investigated the association of each lipid with AD and/or *TREM2* across both brain regions combined by performing a linear model analysis using GLS, as described above, and by adjusting for sex, age at death, number of APOEε4 alleles and post-mortem delay. To further identify brain region-specific associations, an interaction between diagnosis and variant status and brain region was included in each GLS model. To avoid multiple testing issues, an interaction was considered between brain region and AD versus Controls, and between brain region and AD(*TREM2*+) versus AD(*TREM2*−), as these would capture brain region AD-specific or *TREM2*-specific effects. In the presence of an interaction (*P* < 0.05), linear regression analyses were repeated, stratifying by each brain region. As above, we applied a Bonferroni-corrected metabolome-wide statistical significance threshold of *P* < 0.0011.

#### Weighted gene[lipid] co-expression analysis

##### Network construction

To define lipid networks within the BA9 pre-association cortex and HC, we applied WGCNA to the lipid data for each brain region. Lipids were first adjusted for sex, age (at death) and post-mortem delay, separately in each region, and the standardized residuals were used for subsequent analyses. Next, the standardized connectivity (*Z*.*k*) for each sample was computed to identify outliers (Z>|4|). A pairwise correlation matrix using biweight midcorrelations between all lipids was then derived. From this, a weighted, signed adjacency matrix was constructed by raising correlations to a soft thresholding power of 12 for both modules, chosen to meet a scale-free topology threshold of ≥0.8, while maximizing mean connectivity.

Subsequently, the adjacency matrix was transformed into a topological overlap matrix (TOM), representing the network connectivity of lipids. Lipids were then hierarchically clustered into a dendrogram using an average linkage method based on their dissimilarity (1−TOM), and the dendrogram was cut using a dynamic hybrid tree cutting algorithm parameters—minModuleSize = 12 (to allow for smaller modules to be constructed), and mergeHeight = 0.25 (default).

The resulting modules or groups of co-expressed lipids were used to calculate module eigengenes (MEs; or the 1st principal component of the module) for all modules. The ‘gray’ module comprised lipids that were not assigned to any particular module and was therefore dropped from further analyses.

Lipids that are highly connected to their module (termed ‘hub’ lipids) are likely to be functionally important. To identify hubs lipids in each module, the associations between lipids and their assigned module (module membership; kME) were calculated using correlations between lipids and module eigenvalues. Lipids with a kME > 0.70 in each brain region were defined as hubs.

##### Module preservation between BA9 and HC

To investigate whether modules between the two brain regions showed similar coexpression/connectivity and were thus preserved, we utilized the WGCNA ‘modulePreservation’ function. Module preservation and robustness was summarized by reporting Z summary scores, a composite measure of 4 statistics related to density and 3 statistics related to connectivity. The module preservation analysis was applied twice assigning one as the reference dataset and the other as the test dataset. Summary values between 2 and 10 are considered to be moderately preserved (reproducible), while those below 2 are considered not preserved, and those above 10 are considered strongly preserved.^79^

##### Associations between lipid modules and hubs with AD and *TREM2*

Following WGCNA analyses, we sought to investigate the association between lipid modules in each brain region and the following outcomes (i) Post-mortem AD diagnosis (i.e. combining AD(*TREM2*+) and AD(*TREM2*−) donors) versus Control donors. To probe whether any associations of brain lipid modules with AD diagnosis were driven by genetic variation at the *TREM2* locus we then compared brain lipid module levels in (ii) AD(*TREM2*−) brain donors versus Control donors, (iii) AD(*TREM2*+) donors versus Control donors, and finally, (iv) AD(*TREM2*+) donors versus AD(*TREM2*−) donors. Analyses were performed for each brain region separately by regressing the residualized lipid modules generated through WGCNA against the outcomes using linear regression analyses and further adjusting for the number of APOE ε4 alleles. To investigate the association of APOEε4 with lipid modules, separate regression analyses between each module and the number of APOEε4 alleles were additionally fitted. For all module analyses, a Bonferroni-corrected significance threshold was set at *P* < 0.05/4 (Number of modules for each brain region) = 0.0125.

We finally investigated the association of hub lipids, i.e. lipids with kME > 0.70 in modules associated with AD and/or *TREM2*, with the study outcomes. For all hub analyses, a Bonferroni-corrected significance threshold was set at *P* < 0.05/31(Number of hubs) = 0.0016.

Analyses were conducted in RStudio (R version 3.4.2).

## Results

### Donor and sample characteristics

The demographic characteristics of the cohort for each brain region are described in Table 1. Overall, BA9 pre-association cortex tissue was available for 55 donors while HC tissue was available for 47 donors. Although AD donors were generally older, and more likely to be female, there was no statistical difference between age at death, post-mortem delay. The ratio of men to women was similar across groups (*P* = >0.05) (Table 1). Of the 99 annotated lipids 10 were Cer, 16 were SM, 6 were Phosphatidic acids (PA), 22 were PC, including 4 Lyso-phospholipids, 2 were Phosphatidyl-glycerols (PG), 6 were Phosphatidyl-inositols (PI), 13 were PS, 12 were PE and 12 were TGs. Overall, PS levels were higher compared to other lipids (Supplementary Fig. 2).

### Association of lipids with age at death, sex, APOEε4 and post-mortem delay

Most lipids had different levels in BA9 compared to HC. After controlling for multiple testing, 55 out of 99 lipids differed between the two brain regions, 32 of which were lower in the HC and 23 higher in the HC compared to BA9 (Supplementary Table 1).

Following correction for multiple testing, only one lipid, PC 38:2 [PC(38:2)], showed an association with the number of APOEε4 alleles (beta = −0.49, 95% CI = −0.78 to −0.21, *P* = 0.0009). No lipids were associated with sex, age at death or post-mortem delay. Overall, the association of lipids with the covariates were consistent between the two brain regions, in terms of direction of effects, with no interactions present after multiple testing correction (Supplementary Table 1).

### Association of lipids with AD and *TREM2*

**Figure 1:**
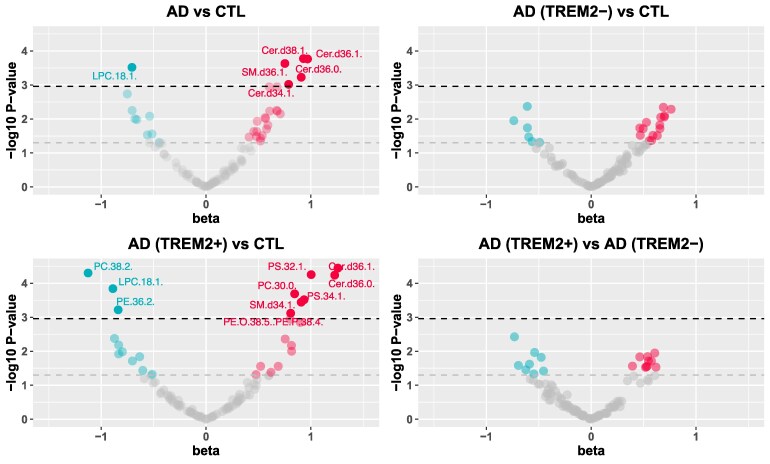
**Volcano plots depicting the association of individual lipids with post-mortem diagnosis and *TREM2* status.** Volcano plot depicting the association of individual lipids (*n* = 99) with post-mortem AD diagnosis (i.e. AD(*TREM2*+) and AD(*TREM2*−) combined versus Control donors) (top-left panel), AD(*TREM2*−) donors versus Control donors (top-right panel), AD(*TREM2*+) versus Control donors (bottom-left panel), and AD(*TREM2*+) donors versus AD(*TREM2*−) donors (bottom-right panel). The *x*-axis represents the change in standard deviations (SD) for each lipid between groups for both brain regions combined following GLS analysis, adjusting for sex, age at death, post-mortem delay and the number of APOE ε4 alleles. The *y*-axis represents the negative logarithm of the *P*-value [−log_10_(*P*-value)]. Annotated lipids are associated with AD or *TREM2* status after Bonferroni correction (*P* < 0.0011). Red dots indicate upregulated lipids at *P* < 0.05, blue dots indicate downregulated lipids at *P* < 0.05, and gray dots indicate non-significant changes (*P* ≥ 0.05). The gray horizontal dotted line indicates the *P* < 0.05 threshold, and the black horizontal dotted line indicates the Bonferroni-corrected significance threshold (*P* < 0.0011). The Bonferroni threshold was derived by dividing the standard significance level (*P* < 0.05) by the number of principal components (*n* = 45) explaining over 95% of the variation in the lipidomic dataset. *N* = 102 (N for BA9 donors = 55, N for HC donors = 47). Abbreviations: AD = Alzheimer’s Disease; AD(TREM2−) = AD donors with no TREM2 rare risk variants; AD(TREM2+) = AD donors with rare TREM2 risk variants; CER = Ceramide; CTL = controls; PA = Phosphatidic acid; PC = Phosphatidyl-choline; PE = Phosphatidyl-ethanolamine; PI = Phosphatidyl-inositol; PS = Phosphatidyl-serine; SM = Sphingomyelin.

Following GLS analyses, 13 lipids were associated with AD and/or *TREM2* status in both regions combined after correction for multiple testing (Fig. 1, Supplementary Table 2). Specifically, six lipids were associated with AD status, five of which were upregulated in AD and one of which was downregulated, with the strongest association being with Ceramide Cer(d38:1) (beta = 0.929, 95% CI 0.46–1.40, *P* = 1.68E−04). Twelve lipids were associated with AD(*TREM2*+) compared to controls, with nine of them being upregulated and three downregulated in AD(*TREM2*+) donors. The strongest association was again with Cer(d38:1) (beta = 1.310, 95% CI 0.78–1.84, *P* = 4.35E−06). We did not observe any difference in lipid levels between AD(*TREM2*−) and control donors or between AD(*TREM2*−) and AD(*TREM2*+) donors after multiple testing correction, although we observed nominal associations for 21 and 17 lipids, respectively.

Overall, five of the six lipids associated with AD status were also associated with AD(*TREM2*+) status compared to controls. Although there were no overlapping lipids with the rest of the group comparisons after correction for multiple testing, we observed overlapping lipids associated with AD and *TREM2* status at nominal *P*-value threshold, as well as a number of associations unique to AD status and unique to *TREM2* status (Supplementary Fig. 3).

### Network analyses

WGCNA analysis identified four modules in each brain region, comprising 16–22 lipids in the BA9 cortex and 14–26 lipids in the HC. A gray module (lipids not assigned to one of the four modules) included the remaining 22 and 17 lipids, respectively.

The four modules identified in BA9 were the turquoise module, comprising mainly of Longer Chain Cer and SMs, and Medium Chain PC lipid species; the yellow module comprising mainly Very Long Chain Phospholipids; the brown module comprising Very Long Chain SMs and Phospholipids, and the blue module comprising mainly TGs.

In the HC, the blue module (corresponding to the BA9 turquoise module) comprised mainly Longer Chain Cer and SMs, and medium chain PC lipid species; the yellow module (equivalent to the BA9 yellow module) comprising mainly Very Long Chain Phospholipids; the turquoise module (corresponding to the BA9 brown and yellow modules) comprising mainly Very Long Chain Phospholipids and Sphingolipids; and the brown module (corresponding to the BA9 blue module) comprising mainly TGs. Module preservation analyses indicated that all four lipid modules showed medium-to-high preservation and reproducibility between the two brain regions (Supplementary Fig. 4).

### Module-level regression analyses

#### BA9

**Figure 2:**
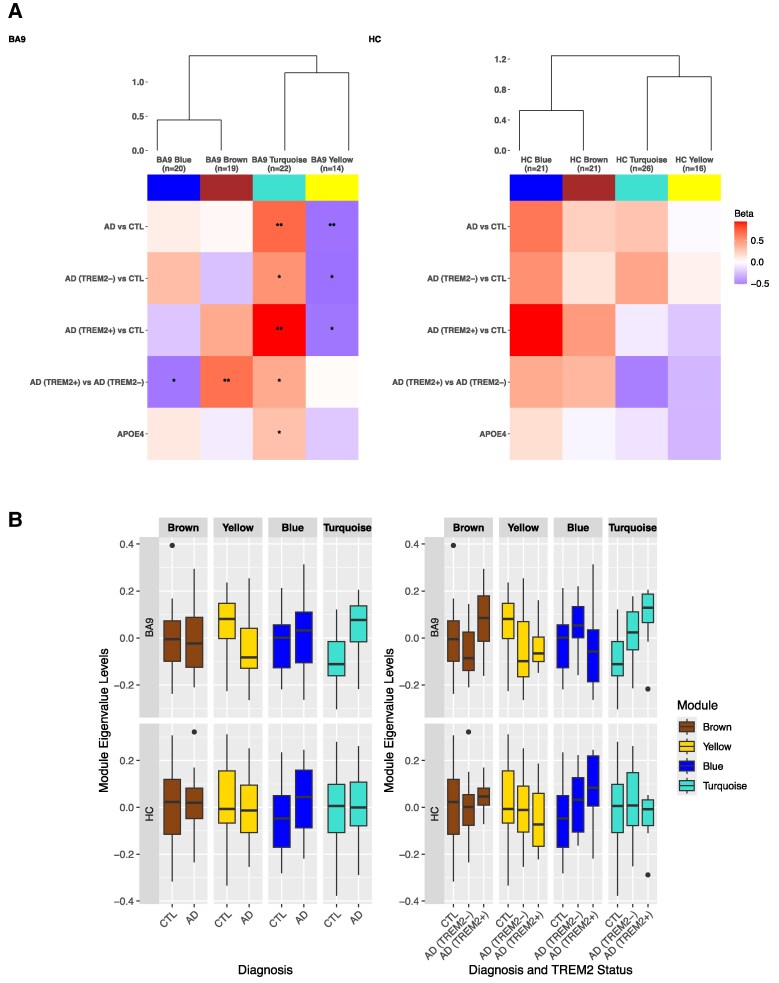
**Associations of lipid module eigenvalues with post-mortem AD diagnosis and *TREM2* status.** (A) Heatmaps depicting the association between module eigenvalues in the BA9 and the HC and (1) Post-mortem AD diagnosis (i.e. AD(*TREM2*+) and AD(*TREM2*−) donors combined versus Control donors), (2) AD(*TREM2*−) donors versus Control donors, (3) AD(*TREM2*+) donors versus Control donors and (4) AD(*TREM2*+) donors versus AD(*TREM2*−) donors, (5) Number of APOE ε4 alleles, following linear regression analyses separately for each brain region. Prior to WGCNA analyses, lipid levels were adjusted for sex, age at death, and post-mortem delay separately in each brain region, and the standardized residuals were used for subsequent network construction. Linear regression analyses assessing associations between residualized module eigenvalues and post-mortem AD diagnosis or *TREM2* status were further adjusted for the number of APOE ε4 alleles. The number of lipids in each module is indicated below the module name. Tile colors reflect the magnitude and direction of the association (β coefficient), with blue indicating a decrease and red indicating an increase in module eigenvalue levels in: AD versus control donors, AD(*TREM2*+) versus AD(*TREM2*−) donors, and in donors with a higher number of APOE ε4 alleles. *P < 0.05; **P < 0.0125. (**B**) Boxplots showing module eigenvalues in AD and Control donors (Left panel), and in AD(*TREM2*−), AD(*TREM2*+) and Control donors (Right panel) for both BA9 and the HC. *N* = 102 (N for BA9 donors = 55, N for HC donors = 47). Abbreviations: AD = Alzheimer’s Disease; AD(TREM2−) = AD donors with no TREM2 rare risk variants; AD(TREM2+) = AD donors with rare TREM2 risk variants; APOE4 = number of APOEε4 alleles; BA9 = Brodmann area 9 pre-association cortex; CTL = controls; HC = Hippocampus. **Module Correspondence**: The BA9 blue module corresponds to the HC brown module; the BA9 brown module corresponds to the HC turquoise module; the BA9 turquoise module corresponds to the HC blue module; the BA9 yellow module corresponds to the HC yellow and turquoise modules.

In linear regression analyses, AD donors had higher turquoise module levels and lower yellow module levels compared to control donors after adjustment for multiple testing (beta = 1.003, 95% CI = 0.42 −1.59, *P* = 0.001 and beta = −0.821, 95% CI = −1.44 to −0.20, *P* = 0.011, respectively) (Fig. 2 and Supplementary Table 3). We next sought to explore whether any associations in lipid module levels observed between AD and control brains were driven by *TREM2* risk variants, by separating AD(*TREM2*−) and AD(*TREM2*+) donors. For the turquoise module, we observed additive AD and *TREM2* effects, whereby levels of lipids in the turquoise module were increased on average by 0.76 SD in AD(*TREM2*−) donors compared to controls (95% CI = 0.13–1.37, *P* = 0.019) and by 1.35 in AD(*TREM2*+) donors compared to controls (95% CI = 0.69–2.01, *P* = 1.467E−04), although only the association between AD(*TREM2*+) donors and controls survived multiple testing correction (Fig. 2 and Supplementary Table 3). Comparing BA9 turquoise module levels between AD(*TREM2*−) donors and AD(*TREM2*+) donors highlighted a modest increase in *TREM2* carriers (beta = 0.601, 95% CI = 0.008–1.19, *P* = 0.047). On the other hand, the decrease in yellow module levels in AD(*TREM2*−) donors relative to controls was similar to that observed in AD(*TREM2*+) donors compared to controls (beta = −0.834, 95% CI −1.52 to −0.145, *P* = 0.019 and beta = −0.03, 95% CI −1.54 to −0.07 *P* = 0.032, respectively), highlighting no additional effect of *TREM2* on lipid levels in the yellow module (*TREM2*-independent associations). None of these associations were significant after correction for multiple testing (Fig. 2 and Supplementary Table 3).

We also observed higher brown module lipid levels and lower blue module levels in AD(*TREM2*+) versus AD(*TREM2*−) carriers (AD-independent associations), although only the association with the brown module survived multiple testing (beta = 0.958, 95% CI = 0.29–1.64, *P* = 0.006) (Fig. 2 and Supplementary Table 3). Finally, we observed a nominal positive association between the turquoise module and the number of APOE ε4 alleles (beta = 0.443, 95% CI = 0.07–0.81, *P* = 0.020).

#### Hippocampus

Linear regression analyses revealed that the direction of effect of the observed associations in the HC was like those observed in BA9, particularly for the HC blue module (corresponding to the BA9 turquoise module). However, the strength of the associations in the HC was weaker to that in BA9 with no nominal associations observed (Fig. 2 and Supplementary Table 3).

#### Association of hub lipids with AD and *TREM2*

We next sought to identify associations between hub lipids that were highly connected (kME > 0.70) in modules associated with AD and/or *TREM2* after correction for multiple testing i.e. the BA9 turquoise, brown and yellow modules. The candidate 31 hubs included 12 lipids from the BA9 turquoise module, 12 lipids from the BA9 yellow module and 7 lipids from the BA9 brown module. The top hub for the BA9 turquoise module was PS [PS(32:1)], followed by longer chain Cer and SM, and PE; the key hub for the BA9 yellow module was PA (PA 36:1) followed by other phospholipids; and the key hub for the BA9 brown module was SM (SM d44:2), followed by other SMs. Since all the modules showed medium-high preservation between the two brain regions, joint brain-area analyses were performed for each lipid using GLS and setting a Bonferroni-corrected significance threshold at *P* < 0.05/31 = 0.0016.

**Figure 3:**
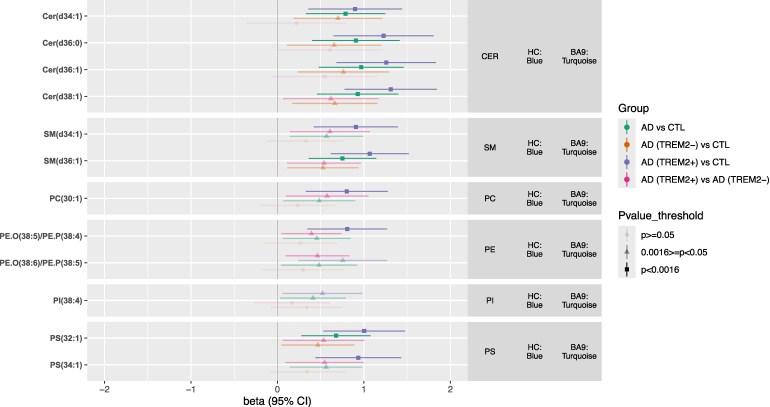
**Forest plot showing associations of hub lipids with post-mortem AD diagnosis and *TREM2* status.** Associations between hub lipids and (1) Post-mortem AD diagnosis (i.e. AD(*TREM2*+) and AD(*TREM2*−) donors combined versus Control donors), (2) AD(*TREM2*−) donors versus Control donors, (3) AD(*TREM2*+) donors versus Control donors and (4) AD(*TREM2*+) donors versus AD(*TREM2*−) donors (BA9 and HC combined) using GLS and adjusting for sex, age at death, post-mortem delay and APOE4 genotype. The columns on the right of the plot indicate the lipid family and module each lipid is assigned to, in each brain region. Lipid levels are standardized to a mean of 0 and SD of 1. Beta coefficients represent the mean difference in standardized lipid levels between two groups. The Bonferroni adjusted threshold is *P* < 0.0016. *N* = 102 (N for BA9 donors = 55, *N* for HC donors = 47). Abbreviations: AD = Alzheimer’s Disease; AD(TREM2−) = AD donors with no TREM2 rare risk variants; AD(TREM2+) = AD donors with rare TREM2 risk variants; APOE4 = number of APOEε4 alleles; BA9 = Brodmann area 9 pre-association cortex; CER = Ceramide; CTL = controls; HC = Hippocampus; PA = Phosphatidic acid; PC = Phosphatidyl-choline; PE = Phosphatidyl-ethanolamine; PI = Phosphatidyl-inositol; PS = Phosphatidyl-serine; SM = Sphingomyelin.

Overall, ten hub lipids were found to be associated with AD and/or *TREM2* status after multiple testing correction (Fig. 3 and Supplementary Table 2). All ten lipids were in the BA9 turquoise module (HC blue module) that consisted of medium chain Phospholipids and longer chain Sphingolipids.

**Figure 4:**
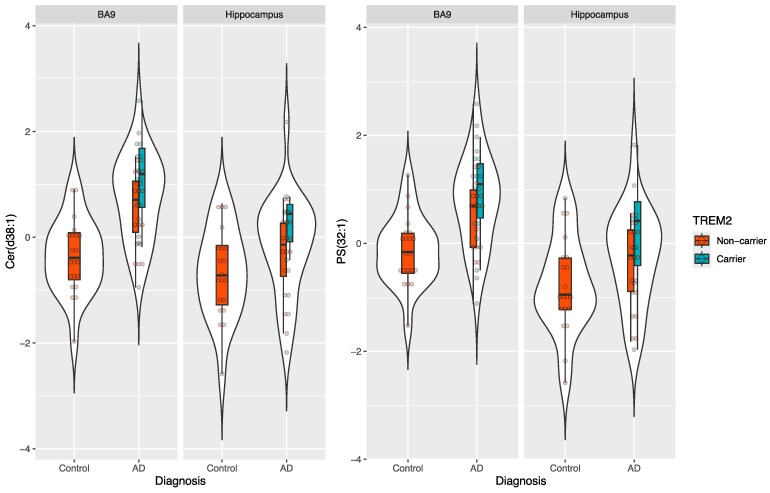
**Violin plots showing the levels of Cer(d38:1) and PS(32:1) lipids in AD(*TREM2*− and *TREM2*+) and control donors, across BA9 and the HC.** Each data point represents lipid levels of Cer(d38:1) and PS(32:1) for each donor. Cer(d38:1) was the top lipid from the turquoise module associated with post-mortem AD diagnosis (i.e. combined AD(*TREM2*+) and AD(*TREM2*−) versus Control donors), as well as with AD(*TREM2*+) versus Control donors. PS(32:1) was the top ‘hub’ lipid in the turquoise module (i.e. the lipid most strongly correlated with the module eigengene). Lipid levels are expressed in standard deviation (SD) units and are unadjusted. *N* = 102 (*N* for BA9 donors = 55, *N* for HC donors = 47). Abbreviations: AD = Alzheimer’s Disease; AD(TREM2−)=AD donors with no TREM2 rare risk variants; AD(TREM2+) = AD donors with rare TREM2 risk variants; BA9 = Brodmann area 9 pre-association cortex; CER = Ceramide; CTL = controls; HC = Hippocampus; PS = Phosphatidyl-serine.

Notably, six of the lipids were increased in AD compared to controls after adjustment for multiple testing. The strongest relationship was with Cer(d38:1); beta = 0.929, 95% CI 0.46–1.40, *P* = 1.68E−04 (Figs 3 and 4), with the remaining associations with three Cer, one Sphingomyelin [SM(d36:1)] and with the BA9 turquoise and HC blue modules key hub Phopsphatidyl-serine [PS(32:1)] (beta = 0.677, 95% CI 0.28–1.08, *P* = 1.14E−03).

We then sought to explore whether any associations between the six hub lipids and AD were driven by *TREM2* risk genotypes. Similarly to module level analyses, GLS analyses for both brain regions highlighted an increase in all BA9 turquoise/HC blue lipid levels ranging from ∼0.47 SD to ∼0.76 SD between AD(*TREM2*−) donors and controls, and an increase in lipid levels ranging from ∼0.80 SD to ∼1.31 SD between AD(*TREM2*+) donors and controls, highlighting on average an increase of ∼0.5 SD in lipids levels between AD(*TREM2*−) and AD(*TREM2*+) . Although the associations between AD(*TREM2*−) and controls were nominal (*P* < 0.05), all six associations between AD(*TREM2*+) and controls were significant after correction for multiple testing (Fig. 3 and Supplementary Table 2). The strongest association was again with Cer(d38:1) (beta = 1.310, 95% CI 0.78–1.84, *P* = 4.35E−06). We also observed four additional lipid hubs, all belonging to the BA9 turquoise/HC blue modules, that were increased in AD(*TREM2*+) carriers versus controls after correction for multiple testing, and which had shown only nominal associations with AD status (Fig. 3 and Supplementary Table 2). These results highlight additive AD and *TREM2* effects for most lipids in the BA9 turquoise and HC blue modules. Finally, we observed some associations with hub lipids in other modules suggesting *TREM2*−independent and AD-independent effects and reflecting the results observed at module level. None of these associations remained significant after adjustment for multiple testing (Supplementary Table 2).

#### Brain region specific association of lipids with AD and *TREM2* variants

Overall, we found associations between brain lipids and end-stage AD with or without a *TREM2* mutation to be consistent between the two brain regions, particularly for the BA9 turquoise/HC blue modules, with overall stronger effects observed for BA9.

There was weak evidence for brain-region-specific associations between *TREM2* and lipids belonging to the BA9 yellow module, where hub Phospholipids were higher in AD donors compared to controls, with no further increase in *TREM2* carriers (*TREM2*-independent associations) (interaction *P* < 0.5) (Supplementary Table 2). We also observed that the levels of hub lipids belonging to the BA9 brown module, particularly SMs, were increased in AD(*TREM2*+) compared to AD(*TREM2*−) donors (AD-independent associations) (interaction *P* < 0.5), although these relationships did not survive multiple testing correction (Supplementary Table 2).

## Discussion

Here, we characterized lipid networks in 102 post-mortem brain samples from 55 Ad and control donors, identifying key lipids associated with AD and potentially impacted by dysfunctional *TREM2*. TREM2 is an established lipid receptor uniquely expressed by microglia in the brain. It is also a risk gene for AD.^19-21,23,27-31^ Four modules consisting of highly correlated lipids were well-preserved between the BA9 pre-association cortex, and HC. Lipid levels in the BA9 ‘turquoise’ module (HC ‘blue’ module) were significantly elevated in post-mortem tissue from AD donors compared to controls. Levels were even higher in AD donors who additionally had a loss-of-function *TREM2*  Ad risk variant. The BA9 ‘turquoise’/HC ‘blue’ module was enriched in longer chain Sphingolipids (Cer and SM), and Phospholipids including PS. Overall, the strongest associations were observed with Cer, whereas the key hub lipid i.e. lipid with the highest number of connections to other lipids in this module, was PS(32:1) in BA9 and HC. These findings further implicate TREM2 in lipid regulation.

PS a major component of cell membranes, is normally confined to the inner cytoplasmic leaflet by transporter proteins flippase and floppase. However, when these enzymes are inactivated during cell death, it becomes exposed on the outer plasma membrane^80^ becoming a phagocytic signal for macrophages including microglia.^81,82^ Local exposure of PS on neurons can impact spine engulfment and thus neural activity.^26^ PS can also impact the structure and function of membrane nanodomains, including cholesterol linked functions.^83^ PS is an established ligand for the TREM2 receptor.^21,23-25^ Our data shows that levels of PS were even higher in *TREM2*  Ad risk variant carriers suggesting a failure by microglia to recognize and clear dysfunctional neurons. Senescent AD microglia are considered poor at degrading aggregate pathologies, leading to aggregate spreading and seeding.^84-87^ Nevertheless, they appear very capable of phagocytosing soma, nucleus and apical dendrites of neuronal corpses and together with astrocytes can fully degrade neurons.^88^ In the course of clearing or partially clearing neuronal corpses in AD brain, insoluble extracellular neurofibrillary ‘ghost tangles’ are left where neurons once resided.^89^ This suggests cell membranes containing PS can be cleared in AD, although whether microglia partially or fully clear neurons in AD in those with a *TREM2* risk variant has not been investigated. In microglia where TREM2 function is compromised, they fail to switch from homeostatic OXPHOS to activated glycolysis^39^ necessary to drive phagocytic function^15,23^ including of myelin debris.^24^ It would be therefore possible that individuals without *TREM2* risk variants wouldn’t develop AD; however, the impact of common SNP variants on downstream TREM2 function (and hence links to AD pathology) is not fully established. It would be interesting to evaluate the number of ‘ghost tangles’ in *TREM2* cases and establish whether membrane remnants remain at these sites to better understand the contribution of TREM2 to neuronal clearance by microglia. A failure to remove apoptotic neurons in AD could lead to the persistence of damaging inflammatory signals from neurons such as HMGB1, DNA and IL-1α which in turn would exacerbate AD pathologies further.^90–93^ It will also be important to evaluate the function of other microglia PS receptors in the absence of TREM2 function. MERTK & Axl, integrin (ITGAV, ITGB3, ITGB5), HAVCR1 & TIMD4, ADGRB1, STAB1 (bridged by C1q), CD300f and LRP1 (bridged by Crt, C1q) are all highly or uniquely expressed by microglia^94^ and may become preferential PS receptors in the absence of TREM2, altering the function of microglia in AD.

PS can exist with differing acyl length and saturation.^95^ PS(32:1) was readily detectable in the two brain areas we investigated and showed the strongest association with AD and *TREM2*, although PS(34:2) was the most abundant in our samples. There were 13 PS molecules detected in our study, four of which belonged to the BA9 ‘turquoise’ module/HC ‘blue’ module, where levels were increased in AD and *TREM2*. It is unknown if there is a preference for different PS species by TREM2 and other PS receptors or why certain PS species are selectively elevated in AD. PS is not only present in neurons, but is also highly abundant in myelin^96^ and other cell types (astrocytes and microglia),^97^ and these could also be a source of the elevated levels we observed in AD and in those with dysfunctional TREM2. Myelin levels could be particularly relevant in light of the raised Ceramide levels we also observed in AD, particularly in those with a *TREM2* risk variant and our finding that TREM2 is central to the co-ordination of oligodendrocyte and microglia genes in AD.^98^

The strongest association with AD and TREM2 dysfunction in our samples was with longer chain Cer. These lipids were important hubs within the BA9 ‘turquoise’ and HC ‘blue’ module, with their kME being slightly lower than that of PS. Cer (galactosylceramide and sulfatide species) are synthesized predominantly by oligodendrocytes in the brain where they are the dominant lipid component of myelin.^96,97,99^ Ceramide species accumulate in AD brains^100^ and leukodystrophies when dysfunctional enzymes required for their metabolism become dysfunctional. This leads to accumulation of intermediate lipid species, notably in microglia lysosomes.^96,101^ Cer are metabolized to Sphingomyelin and vice versa, hence they can be a breakdown product of Sphingomyelin metabolism.^100^ This perhaps explains why Sphingomyelin and Ceramide species were highly correlated and both appeared in the same modules in our study. Together these results, suggest TREM2 mediated microglia function is linked to oligodendrocyte function in AD.

Much effort has consolidated our knowledge of lipid intermediates and the enzymes responsible for their biosynthesis. Acyl chain length and saturation can impact lipid function and intermediates reflect sequential biosynthesis via *de novo* or salvage β-oxidation pathways. Typically, fatty acid chains are categorized as short- (<C5 carbons), medium- (C8–C13 carbons), longer- (C14–C20 carbons) and very long (>C20 carbons), although these categories vary by lipid group,^102,103^ although there isn’t an agreed single universal standard for lipid naming based on chain length. In our study, it was longer chain and not very long chain Cer which had increased levels in AD brain and in those with a *TREM2* risk variant [notably Cer (d38.1)]. Cer play important roles in membrane integrity, and can have proinflammatory and apoptotic function. We and others have previously found longer chain Cer and SMs to be elevated in the plasma of AD/dementia patients and are associated with hippocampal atrophy.^61,103-105^ Consistent with our findings, others have also reported elevated levels in AD brain.^106,107^ A recent study has further reported that Cer mediate the effect of known AD genetic factors such as ABCA7 in AD.^108^ Cer species like the ones reported here have been also implicated in cardiometabolic and cerebrovascular diseases and we have further demonstrated associations with 6-year cardiovascular risk and all-cause mortality in a Type-1-Diabetes cohort.^109^ As both Cer and SMs increase the risk of cardiovascular disease and insulin resistance it has been also suggested that increased cardiometabolic risk could be mediating the association between Cer and dementia risk.^103^ Finally, higher levels of Cer and SMs have been observed in a cuprizone model of myelin damage in mice in which TREM2 is absent.^25^ In fact, sulfatides, which are synthesized from galactosylceramides, which in turn are derived from Cer, are strong TREM2 activators.^110^ We were unable to distinguish sulfatide species in our analysis.

In Nau-Hakola disease where there is complete loss of TREM2 function or its adapter DAP12, lysosomal function in microglia is impaired suggesting the possibility that the increased lipids we observed may be accumulating in microglia thus impacting their response to AD damage.^111^ It will be interesting to establish if microglia from *TREM2*  Ad patients accumulate lipids,^112^ as was recently shown in AD patients carrying APOEε4.^113^ Amyloid deposits also contain lipids including Cer^114^ and this could be an additional source of the elevated Cer we measured. Overall, our results suggest a failure by microglia to recognize damage lipid signals and successfully mobilize to clear damaged myelin and cells in AD leading to accumulation of specific lipids.

Cer can be potent bioactive molecules. High levels of ceramide can cause a myriad of changes culminating in increased damaging reactive oxygen species, through blockade of the respiratory chain, mitophagy and activation of apoptotic factors linked to axonal degeneration and cell death.^100,115,116^ Furthermore, altered membrane lipid composition, particularly galactoceramides, can impact APP processing and amyloidogenic Aβ production^100,115^ and this along with increased TNF-α can impact enzymes that metabolize lipids including those required for Sphingomyelin to Ceramide metabolism, as well as factors linked to apoptosis of oligodendrocytes.^117-119^ In microglia without fully functioning TREM2, a failure to recognize and resolve lipid signals adequately would exacerbate a cycle of damage.

We also found weak evidence for brain region-specific lipid changes. Very long chain SMs and Phospholipids in the BA9 brown module were increased in AD(*TREM2*+) compared to AD(*TREM2*–) donors (AD-independent associations). Hubs within this module also showed nominal associations with *TREM2*, but only in BA9. Similarly, the BA9 yellow module, enriched in very long chain Phospholipids, was decreased in AD compared to control donors, with no further decrease observed in *TREM2* carriers (*TREM2*-independent association). Levels of two hub Phospholipids were increased in AD donors compared to controls in BA9 only, after adjustment for multiple testing (Supplementary Table 2).

Finally, we found that PC, PC(38:2) levels were lower in APOEε4 carriers and also in BA9 in AD donors, particularly those with a *TREM2* risk variant (Supplementary Table 2). PC(38:2) showed borderline association with the BA9 yellow module (kME = 0.694) and was a hub for the equivalent HC yellow module (kME = 0.920). The relationship therefore between *TREM2*, APOE and PC(38:2) warrants further investigation. PC are primarily found in cell membranes including the monolayer encapsulating lipid droplets, a reservoir of neutral lipids and cholesterol esters that swell in AD microglia, particularly in the absence of *TREM2* and in high risk APOEε4 carriers.^25,112,113,120,121^ Their role extends beyond structural integrity, as they are an essential component of lipoproteins that facilitate transport of triacylglycerols/cholesterol to/from cells, a tightly regulated process, with APOE-containing LDL- and HDL-cholesterol particularly implicated in AD.^122-124^ The decrease in brain PC in AD compared to control donors reflects reports by us and others in plasma.^61,125-127^ Additionally, PC like the ones reported here have been positively associated with hippocampal brain volume and negatively with disease progression,^61^ as well as with CSF Aβ1–42.^128^

Although dysregulation of Sphingolipids and Glycerophospholipids in blood and brain has been previously reported by us and others,^61,125-129^ ours is one of the first studies to comprehensively investigate changes in brain lipid levels in AD donors carrying rare *TREM2* risk variants and the first to use two AD-affected brain regions. Others have investigated impacts of *TREM2* on brain gene expression^98^ and metabolites^130^ with findings implicating microglia, oligodendrocyte and endothelial genes, notably those involved in complement and Fcγ receptor function, microglia-associated ribosomal genes and oligodendrocyte genes, particularly proteosomal subunits and amino acid and sphingolipid metabolism and vitamin pathways, respectively. Novotny *et al.*^130^ compared metabolite levels in brain tissue from sporadic AD, familial AD and AD/TREM donors compared to control donors reporting reduced levels of beta-citrylglutamate in AD and AD/*TREM2* brains compared to controls, as well as reduced *α*-tocopherol and CDP-ethanolamine in AD/*TREM2* brains compared to controls brains. Future analyses should investigate associations between lipids identified here, gene expression and protein levels to better understand the molecular processes involved.

Our study has several limitations. The sample size was modest, particularly for the AD(*TREM2*+) group. However, brain tissue was available for most donors from both regions examined, and findings were consistent across these areas. Additional limitations include the use of bulk tissue, which may obscure cell-type-specific lipid alterations, the lack of information regarding the white/gray matter composition of the samples and the fact that these analyses reflect end-stage disease changes. Future studies should focus on analysing lipidomics in larger cohorts of *TREM2* carriers, expand analyses to include blood and CSF—ideally from the same participants- and investigate cell-type-specific changes, Further, associations with individual *TREM2* variants should also be examined, which was not possible in the current study due to limited statistical power. Finally, it is also important to examine lipid changes in brains from ethnically diverse donors to generalize findings.

Overall, our results are consistent with *TREM2* dysfunction interfering with the recognition and clearance of unhealthy neuronal cells, damaged myelin and lipid-containing amyloid. These findings could have great translational potential as they highlight processes to target in future therapeutic strategies.

## Supplementary Material

fcaf452_Supplementary_Data

## Data Availability

Data is available upon reasonable request from the corresponding authors in collaboration with the authors. The code used for these analyses can be found here: https://github.com/Wolfson-PNU-QMUL/TREM2_Lipidomics_Proitsi.
