# Supplementary material for: Alterations in the brain lipidome of Alzheimer's disease donors with rare TREM2 risk variants
Source: Brain Commun. 2026 Jan 21;8(1):fcaf452. doi: 10.1093/braincomms/fcaf452 (PMC12823283; doi:10.1093/braincomms/fcaf452)
Supplement: fcaf452_Supplementary_Data [file fcaf452_supplementary_data.zip › Supplementary_Figures_1-4.docx]

**Supplementary Figure 1.** Heatmap depicting the pairwise Pearson’s correlations between lipids for each brain region.

The colour of the tiles represents the Pearson’s correlation coefficient (r) with red tiles indicating a positive correlation and blue tiles indicating a negative correlation between lipids. .N=102 (N for BA9 donors = 55, N for HC donors = 47).

Abbreviations: CER = Ceramides; SM = sShingomyelins; PA = Phosphatidic acids; PC = Phosphatidyl-cholines; PI = Phosphatidyl-inositols; PS = Phosphatidyl-serines; PE = Phosphatidyl-ethanolamines; TG = Triglycerides. BA9 = Broadmann Areaa 9 Pre-association cortex. HC =. Hippocampus.

**A**

**B**

**Supplementary Figure 2.** Boxplots depicting A) raw lipid levels and B) log-transformed lipid levels prior to quality control in each brain region (BA9 and Hippocampus) separately. N=102 (N for BA9 donors = 55, N for HC donors = 47).

***Abbreviations:*** *BA9= Brodmann area 9 pre-association cortex, CER=Ceramide, HC= Hippocampus. PA=Phosphatidic acid, PC=Phosphatidyl-choline, PE=Phosphatidyl-ethanolamine, PG=Phosphatidyl-glycerol; PI=Phosphatidyl-inositol; PS=Phosphatidyl-serine; SM=Sphingomyelin; TG=Triglycerides.*

**Supplementary Figure 3.** Venn diagram displaying the overlap of nominally significant associations (p < 0.05) between lipids and the following comparison groups: 1) Post-mortem AD diagnosis (i.e., AD ((TREM2+) and AD (TREM2-)) donors combined) vs Control donors, 2) AD (TREM2-) donors vs Control donors, 3) AD (TREM2+) donors vs Control donors, and 4) AD (TREM2+) donors vs AD (TREM2+) donors. Associations were identified using generalized least squares regression for both brain regions combined adjusting for age at death, biological sex, number of APOEε4 alleles, and post-mortem delay. N=102 (N for BA9 donors = 55, N for HC donors = 47).

***Abbreviations:*** *AD=Alzheimer’s Disease, AD(TREM2-)=AD donors with no TREM2 rare risk variants, AD(TREM2+)=AD donors with rare TREM2 risk variants, CTL=controls.*


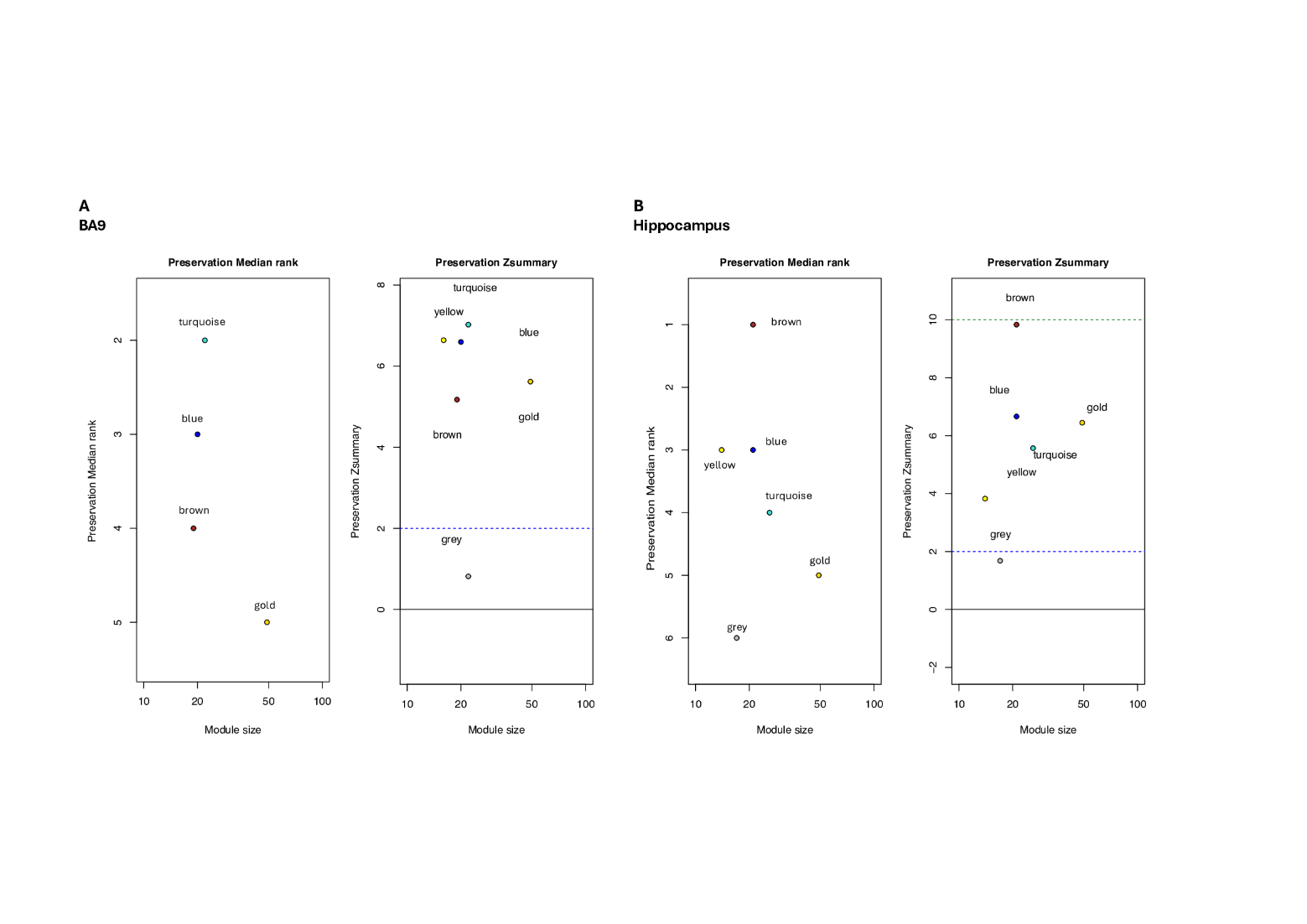


**Supplementary Figure 4.** Module preservation between BA9 and Hippocampus.

Module preservation statistics using A) BA9 as reference and B) Hippocampus as reference. Each coloured dot represents an individual module. The "gold module" is a random sample of lipids from the entire network, serving as a null model to assess the preservation of the actual biological modules. The Z summary score is a composite measure of 4 statistics related to density and 3 statistics related to connectivity. Z summary values between 2 and 10 are considered to be moderately preserved (reproducible), while those below 2 are considered not preserved, and those above 10 are considered strongly preserved. N=102 (N for BA9 donors = 55, N for HC donors = 47).

***Abbreviations:*** *BA9= Brodmann area 9 pre-association cortex; HC= Hippocampus.*
